# The insula in nicotine use disorder: Functional neuroimaging and implications for neuromodulation

**DOI:** 10.1016/j.neubiorev.2019.06.002

**Published:** 2019-06-15

**Authors:** Michael F. Regner, Jason Tregellas, Benzi Kluger, Korey Wylie, Joshua L. Gowin, Jody Tanabe

**Affiliations:** a Department of Radiology, University of Colorado School of Medicine, United States; b Department of Bioengineering, University of Colorado School of Medicine, United States; c Department of Psychiatry, University of Colorado School of Medicine, United States; d Department of Neurology, University of Colorado School of Medicine, United States; e Research Service, Rocky Mountain Regional VA Medical Center, United States

**Keywords:** Insula, Salience network, Nicotine, Tobacco, Cigarettes, Craving, Relapse, fMRI, Neuroimaging, Neuromodulation, Transcranial magnetic stimulation, Human studies, Animal models

## Abstract

Insula dysfunction contributes to nicotine use disorders. Yet, much remains unknown about how insular functions promote nicotine use. We review current models of brain networks in smoking and propose an extension to those models that emphasizes the role of the insula in craving. During acute withdrawal, the insula provides the sensation of craving to the cerebrum and is thought to negotiate craving sensations with cognitive control to guide behavior – either to smoke or abstain. Recent studies have shown that insula processing is saturable, such that different insular functions compete for limited resources. We propose that this saturability explains how craving during withdrawal can overload insular processing to the exclusion of other functions, such as saliency and network homeostasis. A novel signal flow model illustrates how limited insular capacity leads to breakdown of normal function. Finally, we discuss suitability of insula as a neuromodulation target to promote cessation. Given the limited efficacy of standard-of-care treatments for nicotine use disorder, insular neuromodulation offers an innovative, potentially therapeutic target for improving smoking cessation.

## 1. Introduction

Given the serious health problems caused by smoking, it is not surprising that nearly seven in ten smokers want to quit (Babb, 2017). Unfortunately, smoking cessation treatments are largely ineffective, with most attempts to quit failing within the first 24 h. Approximately 80% of patients relapse by six months, despite combined pharmacologic and behavioral therapies (Tobacco, 2008). After six months, only one patient out of ten receiving behavioral therapy alone remains abstinent, and only two patients out of ten receiving combined pharmacological (e.g. nicotine replacement) and behavioral therapy remain abstinent (Stead and Lancaster, 2012). These low success rates underscore the need for more effective interventions and the ability to predict which individuals are most likely to benefit from a given intervention. Achieving these goals requires a better understanding of the neural underpinnings of nicotine use disorder.

A significant advance in our understanding of the neurobiology of smoking behavior was made when Naqvi and colleagues observed that lesions to the insula disrupt the urge to smoke cigarettes (Naqvi et al., 2007). A prospective cohort study extended this findings, reporting that insular damage was associated with increased odds of three-month continuous abstinence (Abdolahi et al., 2015b) and was associated with fewer cravings and withdrawal symptoms compared to non-insular strokes (Abdolahi et al., 2015a, 2017). This finding demonstrated that the insula plays a critical role in smoking maintenance and cravings, and that the region may be a therapeutic target for smoking cessation. (Abdolahi et al., 2015a,b, 2017; Forget et al., 2010; Naqvi et al., 2007; Pushparaj et al., 2013; Suner-Soler et al., 2012). Since lesions of the insula in human and animal studies reduce nicotine craving and consumptions, it implies that insular function contributes to the maintenance of smoking behaviors.

The insula is involved in a wide array of behaviors and functions, including salience attribution, interoception, awareness, affect, anticipation, uncertainty, prediction error, perception, attention, and cognitive processing (Nieuwenhuys, 2012). The insula receives inputs from many sensory modalities and is associated with many cognitive domains (Cauda et al., 2012, 2011). Recent meta-analyses of functional MRI studies suggest that the insula contains two to seven functionally-distinct regions (Cauda et al., 2012; Chang et al., 2013; Deen et al., 2011; Kelly et al., 2012; Kurth et al., 2010). Current insula models suggest that the region plays a role in three broad categories of function (Deen et al., 2011; Uddin et al., 2014). The posterior insula mediates sensorimotor-interoceptive functions, and receives rich afferents from spinothalamocortical pathway carrying nociceptive, thermal, and other interoceptive information (Craig, 2002). The ventral anterior insula is involved in emotional-limbic functions, including peripheral physiological responses to emotional experiences, as measured by heart rate or galvanic skin response (Mutschler et al., 2009; Uddin, 2015; Wang et al., 2018). Ventral anterior insula is associated with the default mode network, which is involved in self-referential processing and includes the precuneus, posterior cingulate gyrus, and ventromedial prefrontal cortex. The dorsal anterior insula is associated with cognitive control functions, such as attention, inhibitory control, and goal-directed cognitive tasks (Dosenbach et al., 2007; Uddin, 2015; Wang et al., 2018). Dorsal anterior insula is associated with the executive control network, which is involved in cognitive control and includes the dorsolateral prefrontal cortex and posterior parietal cortex. Together, the anterior insula receives information from all sensory modalities and domains of brain function, and re-represents this information to form thoughts, feelings, and awareness (Craig, 2002, 2009, 2011).

Despite the functional diversity within insular subunits, several closely-related theories about the functional role of the insula in craving and smoking behaviors have emerged. For example, one theory (Sutherland et al., 2012) suggests that the salience network – consisting of the anterior insula and anterior cingulate – “toggles” between large-scale brain networks, including the executive control network involved in exteroceptive processing and the default mode network involved in self-referential processing. Another theory suggests that the anterior insula is involved in managing predictions against prediction errors (Barrett and Simmons, 2015). While differing somewhat in their models and interpretations, the theories all point to involvement of the anterior insula in processing and balancing of “bottom-up” sensations versus “top-down” cognitive control processes. Because the insula is involved in both craving processing and ability to orient attention to cognitive control and away from visceral cues, modulating the insula may restore cognitive control over craving and withdrawal.

Studies have shown that there is a limit to how much the anterior insula can process. Specifically, evidence shows that the anterior insula is associated with the total capacity or upper functioning limit of cognitive control; the low processing capacity of the insula limits functions such as perceptual encoding (Tombu et al., 2011), response selection (Trautwein et al., 2016), and attentional control (Wu et al., 2019). Insular processing capacity of perceptual and cognitive tasks has been demonstrated to be lower than other brain regions, both by information rate and load (Tombu et al., 2011; Trautwein et al., 2016; Wu et al., 2019). This means that during perceptual and cognitive control tasks, the anterior insula becomes saturated, and it reaches this saturation point earlier than other brain regions. One potential explanation for this saturability or “bottleneck” is that the anterior insula’s concurrent involvement in multiple diverse functions fosters competition amongst functions during a heavy working load for limited resources. For example, the limited processing resources of the insula may be occupied by one function, such as craving, to the exclusion of other functions, such as toggling amongst large scale brain networks.

**Fig. 1.**
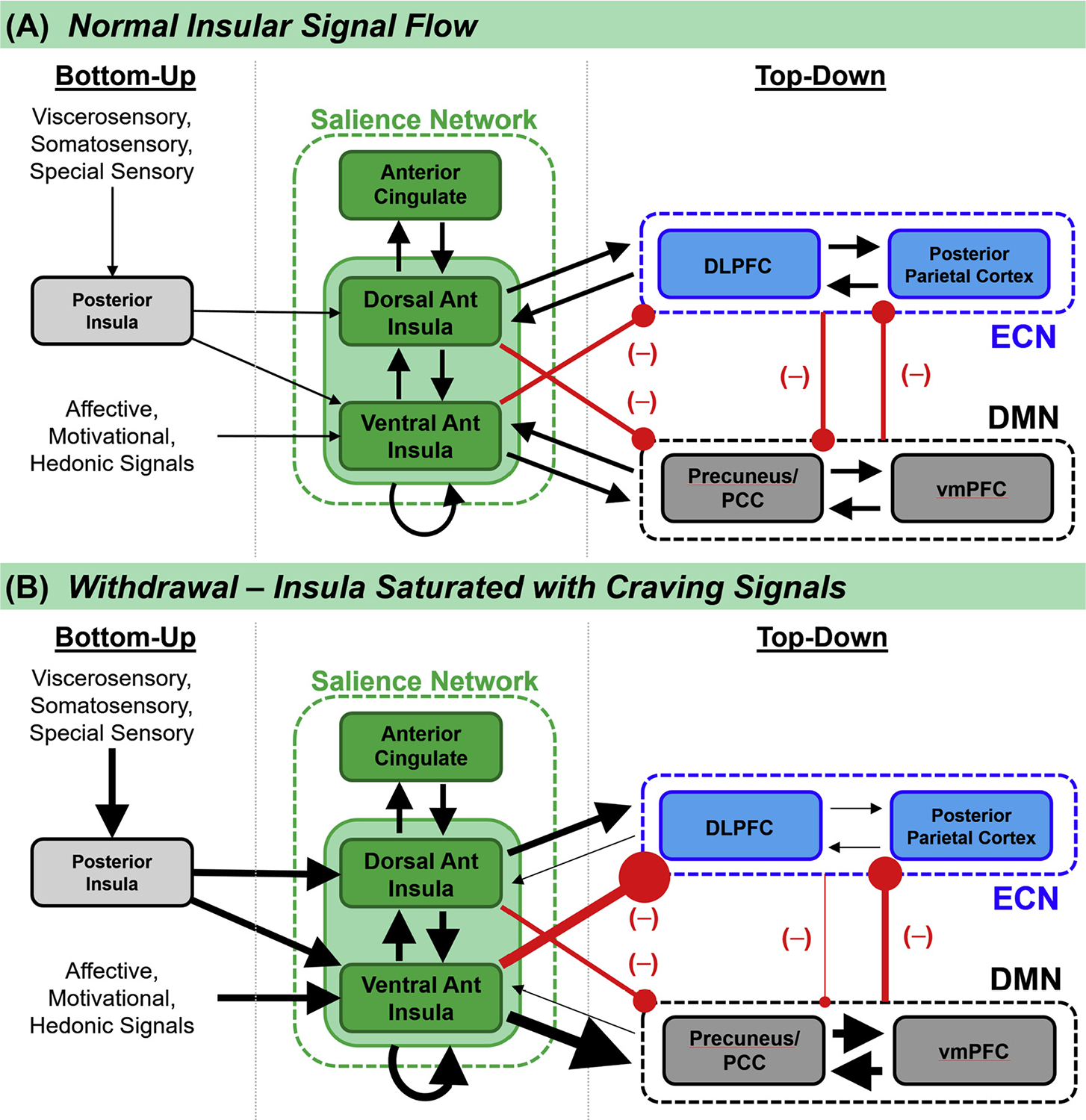
(A) Connectivity-based signal flow diagram (control-system diagram) of anterior insular control of bottom-up versus top-down mechanisms of salience. Information at each node is presumed to be Shannon limited, meaning that there is a finite, tight upper bound on the rate of total information transmission and processing, or the total rates of inputs and outputs (Shannon, 1948). The dorsal anterior insula is involved in processing salience of externally-oriented stimuli and it is connected with the executive control network (an externally-directed system). The ventral anterior insula is involved in processing salience of internally-oriented stimuli and it is connected with the default mode network (an internally-directed system). (B) In the setting of acute withdrawal, craving and withdrawal sensations overload the anterior insula, saturating the limited insular processing bandwidth to the exclusion of normal signal inputs. The insula cannot then negotiate internally-versus externally-directed brain systems. The circuit becomes unstable, and normal homeostasis is lost. Inhibiting the anterior insula may prevent the brain from functional usurpation by craving signals.

The following sections discuss: (1) evidence of insular involvement in nicotine use disorder pathophysiology and (2) possible therapeutic strategies to target the insula for smoking cessation using neuromodulation, which can alter the function of specific brain regions and networks. We review neuroimaging literature on nicotine use disorder, focusing on insular function over different clinical stages. We then review current models of large-scale brain networks in smoking and propose an extension to those models that emphasizes the role of the insula in craving. Compared to models that emphasize toggling (Menon, 2011; Sutherland et al., 2012) or prediction functions of the insula (Chanes and Barrett, 2016; Kleckner et al., 2017), we emphasize that the insula has more limited processing capacity than other brain regions (Wu et al., 2019). During acute withdrawal from nicotine, the insula may become overloaded with sensory craving sensations that prevent it from carrying out its normal processing cognitive control and network homeostasis. We present this extended model using a signal flow diagram (Fig. 1), an approach commonly used in control-systems electrical engineering. We then discuss how modulating this circuitry could provide therapeutic benefit.

## 2. Insular role in nicotine use disorder

### 2.1. Introduction: insular lesions disrupt smoking behaviors

Converging evidence implicates the insula in the maintenance of smoking behaviors and cigarette craving. Naqvi and colleagues (Naqvi et al., 2007) first reported that smokers with cerebrovascular damage to the insula were able to stop smoking without cravings or relapse, suggesting insular function contributes to the urge to smoke. A subsequent, large, prospective study over a one-year period also found that insular lesions in smokers disrupted the desire to smoke and led to abstinence (Suner-Soler et al., 2012). A prospective cohort study (Abdolahi et al., 2015b) conducted in 156 smokers hospitalized for acute ischemic stroke found that insular damage was associated with increased odds of three-month continuous abstinence. Insular damage in the same cohort was also associated with fewer nicotine withdrawal symptoms and cravings compared to those with non-insular strokes (Abdolahi et al., 2015a, 2017). These findings have been corroborated in animal models of nicotine dependence. Insular inactivation in rat models significantly reduced nicotine motivation, nicotine seeking-, and nicotine taking-behaviors, with no effect on food behaviors (Forget et al., 2010; Pushparaj et al., 2013). Both human and animal studies demonstrate that insular lesions disrupt nicotine-seeking behavior behaviors and underscore the need to understand insular function in smokers.

### 2.2. Neuroimaging the insula in nicotine use disorder

**Fig. 2.**
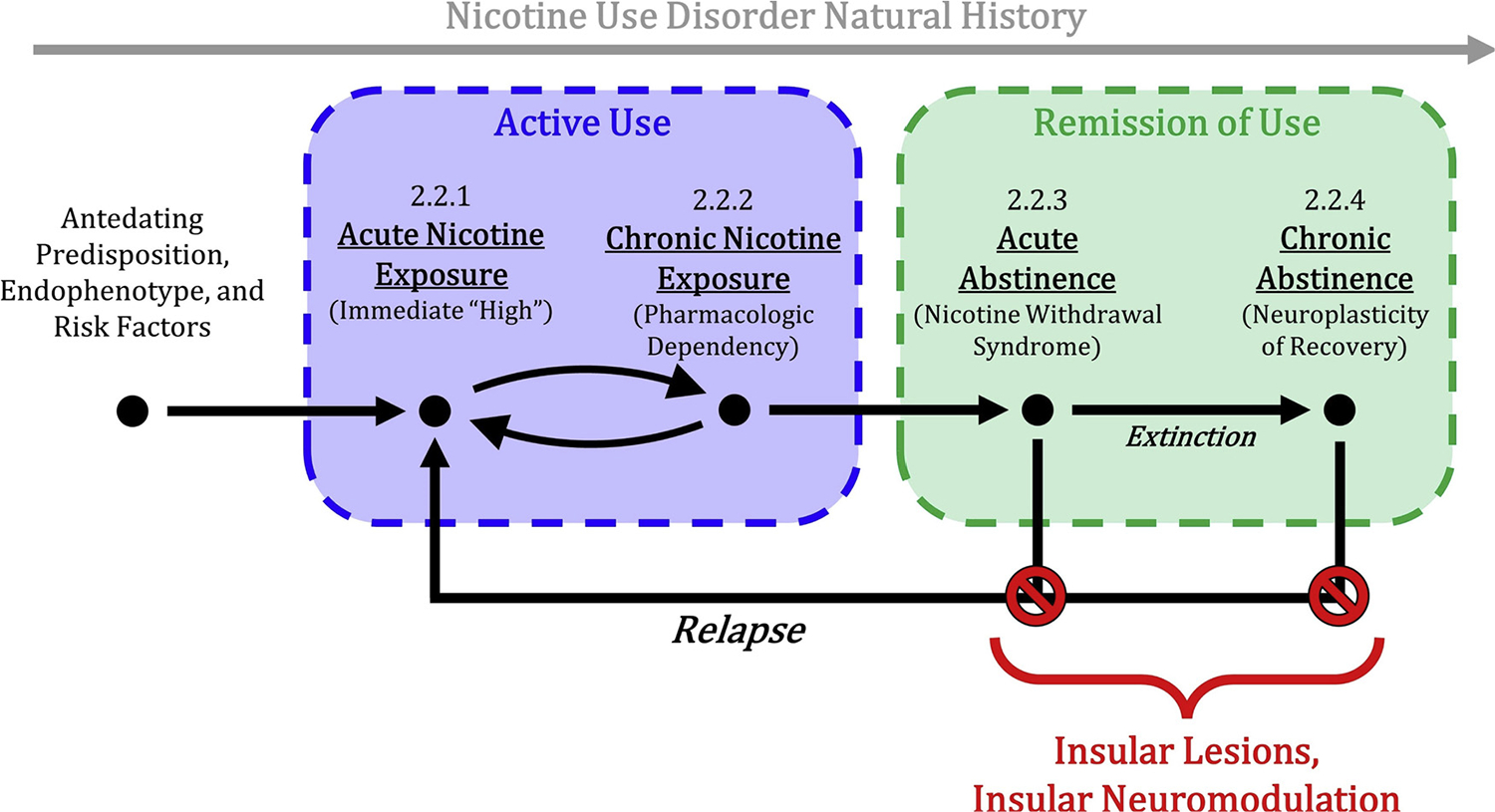
Diagrammatic illustration of the natural history of nicotine use disorder. We attempt to synthesize the neuroimaging literature of nicotine use disorder into four pharmacologically- and behaviorally-informed stages of nicotine use disorder. Acute nicotine exposure (Section 2.2.1) reflects the acute pharmacologic action on neural circuitry. Chronic nicotine exposure (Section 2.2.2) reflects pharmacologic tolerance and dependency and results from repeated nicotine exposure; it manifests as maladaptive changes in reward, salience, and executive control circuitry. Acute abstinence (Section 2.2.3) provides a model for understanding the neural basis of the nicotine withdrawal syndrome and craving. Long-term abstinence (Section 2.2.4) serves as a model of neuroplastic recovery from nicotine use disorder. Associated neuroimaging findings are summarized in Table 1.

Nicotine exposure causes two distinct sets of effects: acute and chronic. Acute effects are short-term changes in behavior and brain function that occur after acute administration. Chronic effects include dependence and tolerance associated with neuroadaptations resulting from repeated exposure to nicotine. We review the neuroimaging literature during four clinical stages of nicotine use disorder and recovery (Fig. 2) focusing on the role of the insula in each stage. First, we review studies of the acute effects of nicotine on neurobiology (Section 2.2.1). Second, we review studies comparing chronic smokers to controls to understand tolerance and dependence (Section 2.2.2). Third, we review studies of nicotine-dependent individuals during acute abstinence to understand the mechanisms of the nicotine withdrawal syndrome and craving (Section 2.2.3). Fourth, we review long-term abstinence as a model of neuroplastic recovery from nicotine use disorder (Section 2.2.4). Finally, we attempt to synthesize the findings from these four stages of the disease into a single model (Section 2.3), summarized in Table 1.

The anterior insula serves several functions, including primary representation of craving sensations in the brain as well as negotiating saliency and attention towards either internally- or externally-directed large scale brain networks. The anterior insula is associated with the total capacity or upper functioning limit of cognitive control; the low processing capacity of the insula compared to other brain regions limits functions including perceptual encoding (Tombu et al., 2011), response selection (Trautwein et al., 2016), and attentional control (Wu et al., 2019). For example, during perceptual decision-making, anterior insula activity increased with greater task load, plateaud earlier than other brain regions, and was associated with the processing capacity of cognitive control (Wu et al., 2019). Another study demonstrated that bottom-up stimulus-reorienting and top-down cognitive control tasks share common anterior insula resource costs (Trautwein et al., 2016), suggesting that these functions compete with each other for limited insular processing resources. This capacity-limit of insular function may apply to acute withdrawal. Withdrawal sensations may overload the anterior insula, preventing it from managing internally-versus externally-directed brain system homeostasis. Previous models have suggested that the salience network, consisting of the anterior insula and anterior cingulate gyrus, “toggles” between the executive control network (consisting of the dorsolateral prefrontal cortex and posterior parietal cortex) and default mode network (consisting of the precuneus, posterior cingulate gyrus, and ventromedial prefrontal cortex) (Sutherland et al., 2012). Here, we extend this model by (1) applying control-systems engineering principles to the model, and (2) emphasizing the saturable, capacity-limited characteristics of the insula to explain neuroimaging findings and motivate novel interventions.

#### 2.2.1. Stage 1: acute nicotine exposure (neural pharmacodynamics)

Like other drugs of abuse, nicotine acts on the brain’s reward circuit and induces dopamine release from ventral tegmental area neurons into the nucleus accumbens and prefrontal cortex (Volkow et al., 2012). Nicotine acts as a ligand at nicotinic acetylcholine receptors (nAChRs). nAChRs are involved in three major circuits: (1) the brainstem’s ascending arousal system of cholinergic neurons that terminates in the ventral tegmental area, (2) the basal forebrain (nucleus basalis of Meynert) cholinergic neurons involved in attention, and (3) fast-acting excitatory post-synaptic potentials in autonomic ganglia associated with visceral sensations (Nestler et al., 2015). By stimulating nAChRs in these circuits, acute nicotine exposure affects arousal, reward, attention, and autonomic regulation.

Functional MRI allows scientists to image *in vivo* the effects of nicotine-induced changes in these circuits. For example, nicotine administration relative to placebo in non-smokers led to decreased default mode network activity (Hahn et al., 2007; Tanabe et al., 2011) and significantly increased local efficiency of connectivity, particularly in right-sided limbic and paralimbic areas (Wylie et al., 2012). A large Activation Likelihood Estimation (ALE) meta-analysis (Sutherland et al., 2015) of acute effects of nAChR agonists on brain activity changes in smokers as measured by fMRI or PET revealed that compared to placebo, administering agonists led to decreased activity in the bilateral anterior insulae (Sutherland et al., 2015). Administering nicotinic agonists also resulted in decreased activity within default mode network regions and increased activity in executive control network regions, possibly explaining cognitive enhancement associated with nicotine administration. This study did not evaluate or control for effects of satiation versus abstinence and heterogeneity of tasks (including resting state), which confounds interpretation, but indicates that insular activity may be altered by nicotine. Relative to placebo, acute nicotine administration leads to decreased anterior insular activity, decreased default mode network activity, and increased executive control network activity, possibly explaining how smoking reduces craving and increases cognitive control.

#### 2.2.2. Stage 2: chronic nicotine exposure in the cigarette-sated state (pharmacologic dependency)

Several studies have compared individuals with a nicotine use disorder to healthy controls during a resting state fMRI scan. One study found that circuit strength between the insula and dorsal anterior cingulate cortex, the two principal nodes of the salience network, was reduced in smokers compared to controls, and smokers with lower circuit strength had greater addiction severity (Moran et al., 2012). These associations were observed when participants were scanned after smoking or after short-term abstinence, suggesting that decreased salience network coherence reflects an effect of nicotine use disorder rather than an acute pharmacologic effect. Salience network coherence has been associated with severity of nicotine use disorder across studies (Bi et al., 2017; Li et al., 2017c; Moran et al., 2012; Wilcox et al., 2017; Zhou et al., 2017). For example, Zhou and colleagues (Zhou et al., 2017) reported that reduced connectivity between the insula and anterior cingulate cortex was associated with increased nicotine use disorder severity. Li and colleagues (Li et al., 2017c) extended these findings, showing that reduced circuit strength between right insula and anterior cingulate cortex was associated with higher number of incongruent errors during a cognitive-control task, implicating this circuit in top-down control of saliency. More importantly, diminished circuit strength between these regions was associated with greater lifetime nicotine consumption. These studies provide evidence that reduced salience network coherence at rest is a marker of chronic nicotine use and reflects addiction severity.

In addition to investigating how insular connectivity relates to addiction severity, studies have examined whether it reflects vulnerability to relapse during future cessation attempts. For example, decreased connectivity between the insula and brain regions involved in cognitive control, including the dorsal anterior cingulate and dorsolateral prefrontal cortices, was associated with greater risk of relapse after attempted cessation (Janes et al., 2010), possibly reflecting a mechanism of reduced cognitive control. Wilcox and colleagues studied 144 individuals with nicotine use disorder during the resting state and reported that decreased circuit strength between the insula and dorsal anterior cingulate was significantly correlated with higher cigarette consumption (Wilcox et al., 2017). After controlling for addiction severity, increased circuit strength between these regions was associated with greater likelihood of successful abstinence. Similarly, a 10-week longitudinal study (Addicott et al., 2015) found that increased insular connectivity to executive control and striatal regions was seen in successful abstainers compared to relapsers. This suggests lower insular connectivity may be associated with relapse vulnerability.

Recent studies have continued to investigate how salience network coherence relates to chronic effects of nicotine use. In one study, circuit strength between the insula and dorsal anterior cingulate cortex was significantly associated with enhanced smoking cue reactivity in areas involved in attention and motor planning, such as the right ventrolateral prefrontal cortex and dorsal striatum (Janes et al., 2015a). Interestingly, the authors reported that insular-anterior cingulate connectivity in smokers remained unchanged across a one-hour period and was unrelated to craving or exhaled carbon monoxide, suggesting that increased salience network coherence may represent a chronic effect of smoking, and may be a neural signature of hypersensitive cue reactivity in nicotine use disorder. Together, these studies suggest that circuit strength between the insula and both (1) anterior cingulate, and (2) regions involved in cognitive control are not only markers of nicotine use disorder, but also a marker of prognosis, since it is associated with ability to quit smoking.

Insula activity in response to emotionally-salient stimuli has been investigated as a biomarker of nicotine dependence. For example, Janes and colleagues. (Janes et al., 2017) studied 23 smokers during a cessation attempt, 10 of whom remained abstinent during a two-week follow up. Relative to successful abstainers, smokers who relapsed demonstrated increased right insular activation in response to cigarette cues, suggesting that this activation predicted likelihood of future use. In another study using a subsample of smokers from the Human Connectome Project dataset, individuals who smoked more cigarettes had greater right anterior insular activation in response to viewing faces expressing negative emotions such as anger (Dias et al., 2016). These studies suggest that greater insula activation in response to both smoking cues and emotional cues may indicate a higher propensity for smoking and relapse. To understand how these observations may relate to propensity to continue smoking, it is important to examine smokers during withdrawal and craving states.

#### 2.2.3. Stage 3: acute abstinence (nicotine withdrawal syndrome)

Acute abstinence is the stage of nicotine use disorder most vulnerable to relapse, and the stage that emphasizes the utility and novelty of our model. Acute abstinence in heavy smokers causes a nicotine withdrawal syndrome, characterized by cigarette craving, hedonic dysregulation, cognitive difficulties, and increased negative affect (Jackson et al., 2015). Craving is a negatively reinforcing aspect of nicotine use disorder and has been shown to increase relapse vulnerability (Ferguson and Shiffman, 2009). In a year-long study of smokers during abstinence, the strength of urges to smoke showed an exponential decline across time, but after a year, over one in three still reported “some urges” to smoke (Ussher et al., 2013). Since the nicotine withdrawal syndrome and craving are persistent, the effects of acute abstinence on brain activity and connectivity could provide insights into nicotine use disorder and its refractoriness to treatment.

Several studies have examined how brain circuits are altered during acute withdrawal, suggesting altered homeostasis amongst salience, executive control, and default mode networks. For example, 24 -h abstinence compared to satiation was associated with weaker negative connectivity between the default mode and salience networks at rest (Lerman et al., 2014). Weaker between-network coupling predicted abstinence-induced cravings and increased activity of default mode network (Lerman et al., 2014). The insula may be involved in causing the altered connectivity observed in withdrawal. To investigate this, Ding and colleagues (Ding and Lee, 2013) studied 21 heavy smokers at rest in cigarette-sated and abstinent conditions, examining effective connectivity between networks and regions of interest. During acute withdrawal, directed connectivity from salience network to default mode network was enhanced and directed connectivity from executive control to the salience network was decreased compared to the sated state. To separate insular versus salience network effects, a small region of interest in the right anterior insula was investigated. In contrast to network level effects, the insula showed significantly increased directed connectivity with salience, default mode, and executive control regions in cigarette abstinence compared to satiation. This suggests that directed information flow from the insula to other brain regions increases in abstinent compared to sated heavy smokers, possibly reflecting increased signaling of cravings. Similar findings have been replicating using independent component analysis during resting state and psychophysiologic interactions during craving cue tasks (Hobkirk et al., 2018; Huang et al., 2014; Moran-Santa Maria et al., 2015). These findings suggest that withdrawal leads to (1) increased anterior insular signaling to brain networks involved in salience, executive control, and self-referential thought, possibly reflecting craving, and (2) reduced coupling between these three major networks. Together, the findings suggest that in acute withdrawal, the insula is overloaded with the transmission of craving signals, with no available resources to maintain a normal balance between large scale networks.

Neural responses to cigarette cues in 116 smokers abstinent for ≥3 h were examined using a psychophysiological interaction centered on a dorsal anterior insular seed to infer effective connectivity (Claus et al., 2013). Smoking cues compared to neutral cues caused stronger signaling from the insula to multiple nodes, including amygdala, somatosensory cortex, orbitofrontal cortex, and striatum. This may indicate that the anterior insula contributes to the initial evaluation of cigarette cue value, interoceptive processing of withdrawal symptoms, and engagement of motor circuits in preparation for drug-seeking behavior.

An individual’s beliefs about what will happen following nicotine administration have been shown to modulate the effects of acute withdrawal. Gu and colleagues (Gu et al., 2016) studied 24 overnight abstinent smokers who performed a sequential reward learning task immediately after a cigarette-smoking intervention. Smokers received either a 0.6 mg nicotine cigarette or a de-nicotinized cigarette. To alter drug expectancy, smokers were told that the cigarette either contained “nicotine” or “no nicotine”. All participants completed all four conditions. Activation of the ventral anterior insula during reward learning occurred only when smokers received a cigarette with nicotine and were told that it contained nicotine. Higher insular activity was associated with greater craving magnitude. This suggests that the anterior insula is not only involved in interoceptive processing, but that anterior insula processing of craving and reinforcement learning is modulated by drug expectancy, presumably through cognitive control influences.

#### 2.2.4. Stage 4: chronic abstinence (neuroplastic recovery)

It is unclear whether neural function returns to healthy levels following long-term abstinence, or if the differences associated with nicotine use disorder persist. Although chronic nicotine exposure results in upregulation of nicotinic acetylcholine receptors throughout the brain (Breese et al., 1997; Gentry and Lukas, 2002), former smokers exhibit nicotinic acetylcholine receptors concentrations similar to non-smokers (Breese et al., 1997), suggesting that upregulation is reversible. Evidence of neuroplastic recovery is suggested by behavioral changes during chronic abstinence. Measures of impulsivity have been shown to be elevated in active smokers, but former smokers show levels similar to never-smokers (Bickel et al., 1999). Studies of ex-smokers thus may provide important insights into mechanisms of successful smoking cessation.

Several studies have examined insula connectivity in chronic abstinence. Zanchi and colleagues (Zanchi et al., 2015) studied non-smokers, active smokers, and ex-smokers during a craving-cue task during an fMRI scan. Ex-smokers with greater right anterior insular activity in response to cigarette cues had higher lifetime nicotine consumption, and ex-smokers demonstrated decreased circuit strength between the right anterior insula and anterior cingulate compared to non-smokers, but ex-smokers and active smokers did not differ. This suggests that insular function may not completely recover in long-term abstinence, possibly reflecting a mechanism of persistent craving. Another fMRI study (Nestor et al., 2011) of smokers, ex-smokers, and healthy controls used an attentional bias paradigm with neutral cues, emotionally-evocative cues, and smoking cues. Across all cue conditions, ex-smokers exhibited significantly greater activation in the right anterior insula compared to active smokers and controls. In a separate experiment employing a go/no-go paradigm to investigate motor response inhibition and cognitive error monitoring, ex-smokers had significantly greater error-related activation than both controls and smokers in the left insula. Taken together, these results suggest that heightened insular monitoring of cues and errors contribute to the successful maintenance of abstinence. Higher anterior insular activity in ex-smokers compared to healthy controls may reflect a persistent hypersensitivity to smoking cues.

### 2.3. Putting it all together: unified models of the role of the insula in nicotine use disorder pathogenesis

One of the primary functions of the anterior insula is salience detection (Seeley et al., 2007), such as identifying stimulus features that stand out, are learned, or are instinctually important. This theory holds that the insula selects stimuli from a continuous stream of internal and external sensory inputs for additional processing. Another theory is that the anterior insula serves as the “apex of a predictive cortical hierarchy” that spans all sensory systems (Barrett and Simmons, 2015; Chanes and Barrett, 2016), using numerous streams of prediction errors to fine-tune and send prediction signals throughout the cortex, which are then used to determine prediction-errors. The insula is also involved in response selection and craving processing. It is unique amongst cortical areas in that it contains sequential yet overlapping maps from all exteroceptive and interoceptive senses (Craig, 2009, 2011). These higher-order maps are successively re-represented from posterior insula to middle insula to anterior insula, progressively acquiring additional sensory, interoceptive, learning, reward, and cognitive signals along the way. The anterior insula then provides a single cortical representation of how an individual is feeling at a given time: the “global emotional moment” or “cinemascopic awareness” (Craig, 2009, 2011). However, as discussed in the Introduction, the anterior insula has a limited capacity to process information such as perceptual tasks with varying cognitive loads (Wu et al., 2019). From this perspective, the anterior insula is a saturable neural node - it cannot receive and process more than a certain amount of information at a given time. These models suggest broad involvement of the anterior insula in craving, polysensory processing, and negotiating sensory input versus cognitive control mechanisms of salience. One function may outcompete others for insular processing resources, for example, in the case of pathologic overstimulation of craving signals.

The triple network hypothesis (Menon, 2011) has been applied to explain the insula’s role in nicotine use disorder (Sutherland et al., 2012). It suggests that the insula, along with the anterior cingulate cortex, serves as a toggle, directing brain function towards internal or external stimuli, in order to maintain homeostasis of cognitive resources and guide goal-directed behavior (Sutherland et al., 2012). Internal focus is reflected by greater default mode network (endogenous-oriented) activity, whereas external focus is reflected by greater executive control network (exogenous-oriented) activity. In this hypothesis, chronic nicotine use disrupts the insula’s toggling function by redirecting resources toward endogenous processing in the default mode network. Lerman et al. (2014) provided supporting evidence for this model, reporting decreased between-network coherence amongst salience, default mode, and executive control networks in acute abstinence compared to satiety. They reported that weaker between-network coupling predicted abstinence-induced cravings and less suppression of default mode activity during performance of a subsequent working memory task, possibly reflecting a mechanism of cognitive and attentive impairments commonly observed during the nicotine withdrawal syndrome.

**Fig. 3.**
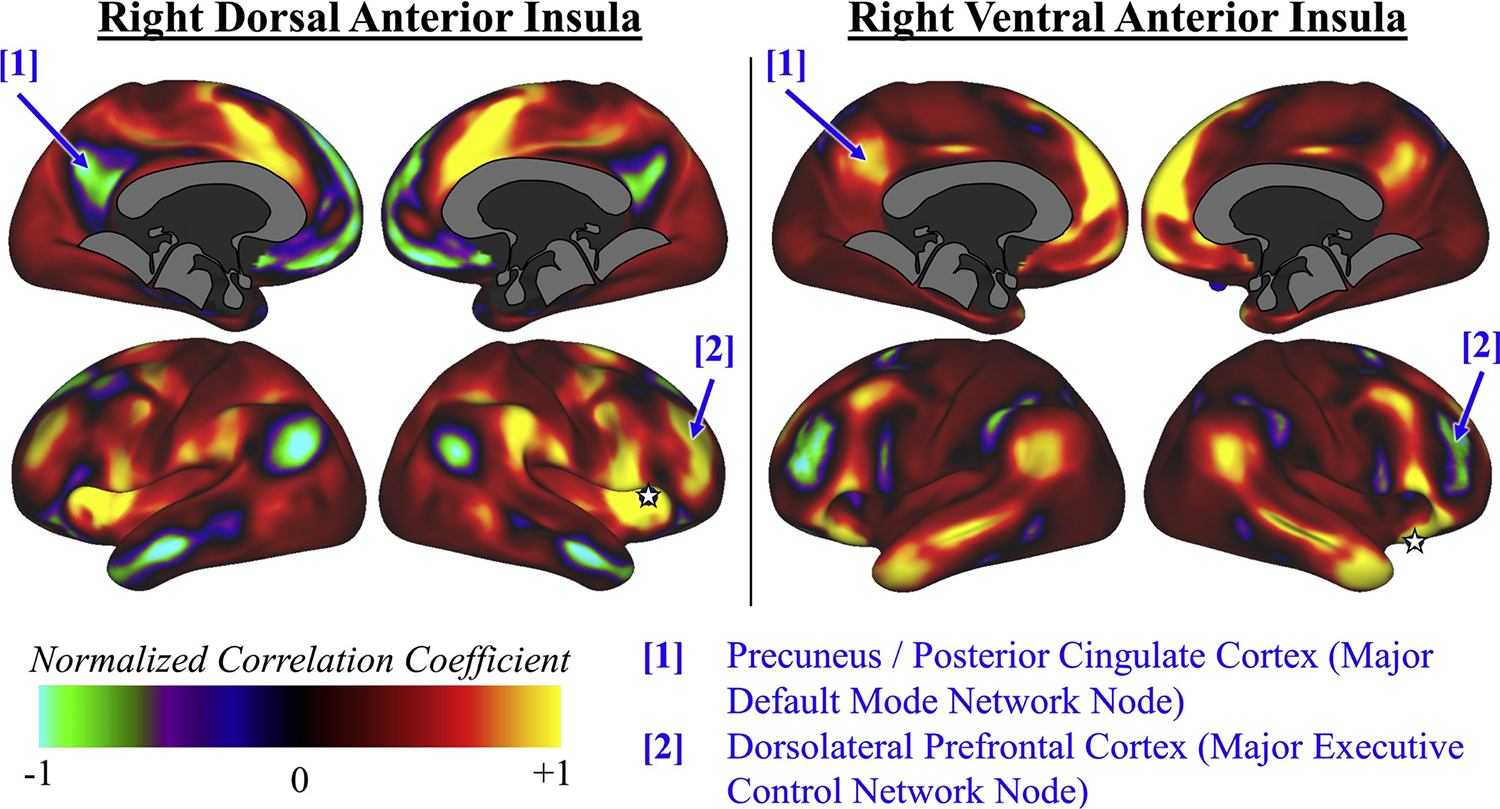
Differential resting-state connectivity of the dorsal (left) and ventral (right) right anterior insula, using the Human Connectome Project Connectome Workbench (n = 1206), uncorrected. Dorsal right anterior insula is strongly connected with frontoparietal regions involved in executive control; in contrast, ventral right anterior insula connectivity is strongly connected with default mode network regions involved in internal feelings and self-referential processing. Note that the externally-directed system (dorsal anterior insula and executive control network) and internally-directed system (ventral anterior insula and default mode network) are inversely correlated, consistent with mutual inhibition (see Fig. 1).

As an extension to these models, we propose (Fig. 1) that total insular function is bandwidth-limited, and that this limits the insula’s ability to represent craving, negotiate salience, and toggle between networks under a heavy working load. The concept that the insula directs attention towards the most relevant stimuli – internal or external – explains both cognitive changes and functional connectivity findings of acute nicotine ingestion (Section 2.2.1), nicotine satiety in dependence (Section 2.2.2), and nicotine withdrawal syndrome (Section 2.2.3). The insula becomes overburdened with conscious awareness of craving and withdrawal symptoms during acute abstinence. Saturation of insular resources with craving processing then impairs its ability to coordinate normal coupling mechanisms between the three large scale brain networks (Fig. 3).

The evidence suggests that insular function is disrupted compared to healthy controls across all stages of nicotine use disorder (Table 1). Salience network coherence between insular and anterior cingulate nodes is particularly important for craving-induced behaviors, reflects disease severity, and has prognostic value. Since insula activation is associated with craving and is higher for smokers experiencing withdrawal, reducing insula activation may be a strategy to reduce relapse.

## 3. Implications of insular role in nicotine use disorder on neuromodulatory therapeutic development

Since current treatments have limited efficacy, new treatment strategies are needed. One possibility is a targeted circuit-node approach, aligned with current understanding of the underlying pathology. A neurocircuit-based approach may improve cessation by modulating key neuroanatomic nodes such as the insula. A promising candidate for this approach is neuromodulation with transcranial magnetic stimulation.

### 3.1. Therapeutic neuromodulation in animal models of nicotine use disorder

Neuromodulation has been examined as a tool for reducing nicotine use in animals. (Forget et al., 2010) Insular inactivation via intracranial GABA agonist microinfusion in nicotine-dependent rats significantly reduced nicotine motivation, nicotine seeking-, and nicotine taking-behaviors, with no effect on food behaviors (Forget et al., 2010). These findings were confirmed using an alternative lesioning method, bilateral insular deep brain stimulation, in a rat model of nicotine dependence (Pushparaj et al., 2013). Kutlu et al. (2013) showed that locally infused D_1_ (but not D_2_) antagonists into the rostral anterior insular cortex decreased rats’ nicotine self-administration acutely by more than 50%, with repeated D_1_ antagonist infusions resulting in continued decreases in nicotine consumption. The cause-and-effect relationship between decreased D_1_ activity in the insula and decreased nicotine self-administration suggests that midbrain dopaminergic afferents onto the anterior insula are critically involved in promoting and maintaining nicotine dependence (Kutlu et al., 2013). Disrupting this insular mechanism may lead to diminished nicotine consumption.

### 3.2. Therapeutic neuromodulation in humans with nicotine use disorder

Transcranial magnetic stimulation (TMS) is a technique that may be used to modulate insula function. TMS works by placing electrical coils near the scalp and running a current through it to induce rapid, transient, focal magnetic fields. These transient magnetic fields cause electromagnetic induction in underlying neural tissues that alter neural transmembrane potentials and neural activity. Applying a sequence of TMS pulses can either facilitate or inhibit neuronal excitability, depending upon pulse parameters and stimulation site. Based on studies of the corticospinal motor tract, low-frequency (≤ 1 Hz) repetitive TMS is inhibitory and high-frequency (≥ 5 Hz) repetitive TMS is faciliatory, with aftereffects closely paralleling long-term depression and long-term potentiation mechanisms of neuroplasticity (Hoogendam et al., 2010).

Several studies have investigated whether TMS targeting the dorsolateral prefrontal cortex affects cigarette craving and consumption. Most studies focus on excitatory TMS of the dorsolateral prefrontal cortex in smokers (Amiaz et al., 2009; Chang et al., 2018; Li et al., 2013), likely because of its efficacy in major depressive disorder (Brunoni et al., 2017; Milev et al., 2016) and relative accessibility of this region as a superficial target site compared to other, deeper structures. Most studies rely on scalp landmarks for targeting (Amiaz et al., 2009; Dinur-Klein et al., 2014; Kozak et al., 2018). Some studies have used neuroimaging for subject-specific neuronavigation (Pripfl et al., 2014; Sheffer et al., 2018; Trojak et al., 2015), and few studies have demonstrated target engagement through neuroimaging (Chang et al., 2018; Li et al., 2017a, b).

Most of these studies demonstrate decreases in craving and cigarette consumption after dorsolateral prefrontal cortex TMS (Amiaz et al., 2009; Chang et al., 2018; Dinur-Klein et al., 2014; Trojak et al., 2015); however, the evidence is mixed. For example, some studies demonstrate decreased craving without a change in cigarette consumption (Wing et al., 2012), while others demonstrate decreased cigarette consumption without a change in craving (Dieler et al., 2014; Johann et al., 2003). Thus, there is room for improvement. Inhibiting the craving urges from the cortical source, which becomes overloaded with craving signals to the detriment of cognitive control, may be more effective. Human stroke and animal neuromodulation studies reviewed here implicate a crucial role of the insula in sustaining craving sensations and nicotine-consuming behaviors. Towards this end, our laboratory is conducting a randomized, sham-controlled clinical trial (NCT02590640) in active smokers to investigate the efficacy of insular inhibitory neuromodulation to reduce cigarette craving in acute abstinent smokers.

**Fig. 4.**
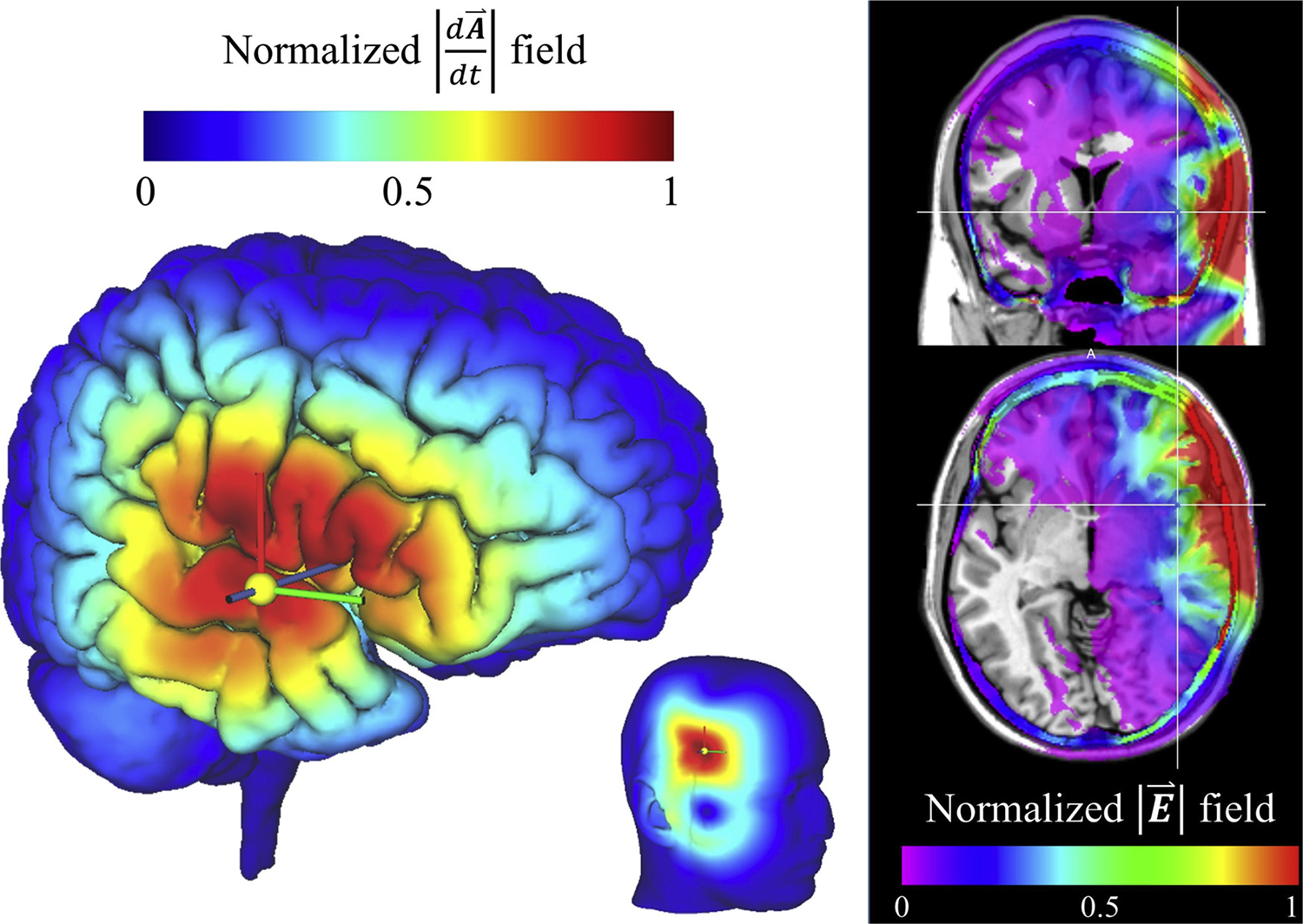
Finite element model results of the predicted electromagnetic field produced by a standard planar transcranial magnetic stimulation 70 mm figure-of-eight coil targeting the right anterior insula. Predicted current flux density (left) and normalized absolute value of the electric field (right) illustrating the pattern of energy deposition. Maximum energy is deposited in the scalp, superficial soft tissues, and cerebrospinal fluid due to high tissue conductivities. This illustrates the difficulty in targeting deep structures, such as the insula or anterior cingulate cortex. This is consistent with model results of insular targeting reported by Pollatos and colleagues (Pollatos and Kammer, 2017). Created using SimNIBS version 2.1.1 (Thielscher et al., 2015) with right anterior insular target [36, 10, −6] in MNI space and default parameters (unpublished).

One of the major technical limitations of the application of TMS to the insula is the limited spatial depth of electromagnetic induction and its inverse relationship with focality. Across different coil geometries, stimulation of deeper brain targets necessitates greater electrical field spread and reduced focality (Deng et al., 2013). This tradeoff between electric field depth and focality poses important physical challenges to stimulating the anterior insula. One study applied continuous TMS to the right anterior insular cortex and control regions (occipital and somatosensory cortices) in healthy volunteers using a superficial (i.e., planar figure-of-eight) coil (Pollatos et al., 2016). Their results suggested that inhibiting the right anterior insula was associated with a significant decrease in cardiac and respiratory interoceptive accuracy (measured by a heartbeat counting task). The targetability of the anterior insula using a conventional coil has been debated (Coll et al., 2017; Pollatos and Kammer, 2017). Investigators noted that by using this approach the anterior insula receives about 25–35% of the maximum cortical energy deposition and greatest deposition in the over-lying frontal and temporal opercula (Fig. 4). The results suggest that subthreshold levels of stimulation can affect neural function and that choice of coil geometry is important.

Recently, a family of coil designs called Hesed (H) coils have been developed to achieve deep brain neuromodulation at the expense of a wide, relatively non-focal treatment field. These coils provide near-complete stimulation of the lateral frontal lobes. Dinur-Klein and colleagues (Dinur-Klein et al., 2014) randomized 115 heavy smokers to 13 daily treatments of high-frequency, low-frequency, or sham TMS using an H-coil designed to target the bilateral dorsolateral prefrontal cortex, ventrolateral prefrontal cortex, and insula. Smoking was measured by participants’ self-report and urine cotinine levels. High-frequency TMS, but not low-frequency or sham TMS, resulted in a 44% reduction in smoking at 3 months and 33% reduction at six months. There was no significant difference in self-reported craving, suggesting that these effects may reflect enhanced cognitive control through dorsolateral prefrontal cortex augmentation rather than reduced incentive salience or reduced sensation of cigarette craving. Malik et al. (2017) applied excitatory and inhibitory TMS to the bilateral insula and surrounding cortical opercula using the H-coil in eight healthy participants. Synaptic effects were measured using PET with a dopamine agonist tracer. They demonstrated that inhibitory (1 Hz) TMS compared to sham and excitatory (10 Hz) TMS significantly decreased dopamine concentrations in the substantia nigra and sensorimotor striatum, suggesting a mechanism of action for TMS. In both these studies reporting insular modulation, the investigators could not definitively confirm that the insular cortex was stimulated, although future studies using fMRI could address this question.

Neuromodulatory methods not only include TMS, but also include transcranial direct current stimulation (tDCS) and deep brain stimulation (DBS). tDCS involves applying an electrical current to the brain between two electrodes, which affects neural tissues within the path of least electrical resistance. tDCS has been used to target the dorsolateral prefrontal cortex in smokers with reduction in cue-induced cravings; however, the non-focal nature of this method limits its utility in targeted, neuroanatomically-driven neuromodulation (Salling and Martinez, 2016). DBS, on the other hand, involves surgically implanting a stimulating electrode into target brain tissue. DBS targeting the ventral striatum in smokers has been reported in only one study, which reported higher rates of successful cessation compared to unaided smoking cessation in the general population (Kuhn et al., 2009). However, surgically-placed deep brain stimulation is invasive and practically limited by ethical considerations.

In summary, applications of non-invasive methods of brain stimulation in nicotine addiction are promising but limited by their lack of spatial specificity and depth of targetability. Modulation of dorsolateral prefrontal cortex has been shown to decrease cravings and improve abstinence rates. While the anterior insula has empiric support in the basic science literature and may result in stronger treatment responses, few studies have targeted this region. Evidence implicating the insula in craving sensations, smoking behaviors, and relapse suggests that modulation of the insula is an important area of future work. To date, studies in smokers have primarily focused on augmenting brain regions involved in cognitive control and have neglected the possibility of inhibiting bottom-up brain regions directly responsible for craving. Future studies are needed to define optimal targets, paradigms, and patient population.

## 4. Conclusion

The insula is functionally heterogeneous, with distinct patterns of connectivity with large-scale brain networks associated with numerous functions and behaviors. Animal models and human lesion studies suggest that the insula is necessary for the maintenance of nicotine-seeking behaviors and nicotine-taking behaviors, likely through nicotine craving. However, studies have shown that the insula is a saturable node of information processing. We propose that this saturability explains how insula functions at each stage in nicotine use disorder. Craving during withdrawal can overload insular processing to the exclusion of other functions, such as saliency and network homeostasis. We propose a novel signal flow model to illustrate how this limited capacity of the insula leads to breakdown of normal function during acute nicotine withdrawal. This model also illustrates a mechanism through which insular neuromodulation may promote abstinence. Given the limited efficacy of standard-of-care treatments for nicotine use disorder, modulation of this region may be a promising strategy for the next generation of treatments by offering what has been previously unavailable: a targeted, neuroanatomically-driven therapy.

## Figures and Tables

**Table 1:** Stage-by-stage analysis of large-scale brain network neuroimaging findings and associated role of the insula across different stages of the natural history of nicotine use disorders. The neuropharmacologic mechanisms associated with each disease stage are listed in italics.

Disease Stage *Drug-Induced Mechanism*	Neuroimaging Findings	Role of the Insula
**Acute Nicotine Exposure**	Anterior insula activity (Sutherland 2015) Default mode network activity (Tanabe 2011, Sutherland 2015) ECN activity → increased attention (Sutherland 2015) SN-DMN-ECN connectivity (Lerman et al., 2014)	Represents sensations associated with nicotine reward across all sensory modalities Allows redirection of resources away from interoceptive (DMN) and towards exteroceptive (ECN).
*Neural Pharmacodynamics*		
**Chronic Nicotine Exposure**	Insulo-cingulate connectivity Associated with: FTND, incongruent errors on Stroop task, lifetime nicotine consumption (pack-years), future relapse (Janes et al., 2010; Li et al., 2017c) Not associated with craving or recent smoking → durable feature of dependency Associated striatal adaptations, progressively ventral to dorsal reflecting habit formation Insular connectivity with ECN and striatal regions associated with successful abstainers (Addicott et al., 2015)	New homeostatic set point for sensations associated with nicotine-induced reward Cigarette-related memory retrieval (Janes et al., 2015b) Increased cue-related insular activity is associated with future use (Janes et al., 2017)
*Pharmacologic Dependency*		
**Acute Abstinence**	SN-DMN-ECN connectivity (Lerman et al., 2014) Granger causality from insula to SN, DMN, and ECN → increased craving signaling (Ding and Lee, 2013) Right Anterior Insula – DMN connectivity, associated with craving magnitude (Moran-Santa Maria et al., 2015)	Represents sensations associated with cravings across all sensory modalities (Abdolahi et al., 2015a, b, 2017) Insula becomes saturated with craving signals → inability to manage executive control functions Loss of insular ability to suppress DMN activity (Lerman et al., 2014)
* Nicotine Withdrawal Syndrome*		
**Chronic Abstinence**	Right anterior insular activation in response to cue exposure, associated with lifetime nicotine consumption (Nestor et al., 2011; Zanchi et al., 2015) Salience Network Coherence → possible irreversible phenotype (Zanchi et al., 2015)	Long-lasting hypersaliency of nicotine cues
* Neuroplastic Recovery*		

## References

[R1] Abdolahi A, Williams GC, Benesch CG, Wang HZ, Spitzer EM, Scott BE, Block RC, van Wijngaarden E, 2015a. Damage to the insula leads to decreased nicotine withdrawal during abstinence. Addiction 110, 1994–2003. 26347067 10.1111/add.13061 PMC4644476

[R2] Abdolahi A, Williams GC, Benesch CG, Wang HZ, Spitzer EM, Scott BE, Block RC, van Wijngaarden E, 2015b. Smoking cessation behaviors three months following acute insular damage from stroke. Addict. Behav 51, 24–30. 26188468 10.1016/j.addbeh.2015.07.001 PMC4558299

[R3] Abdolahi A, Williams GC, Benesch CG, Wang HZ, Spitzer EM, Scott BE, Block RC, van Wijngaarden E, 2017. Immediate and sustained decrease in smoking urges after acute insular cortex damage. Nicotine Tob. Res 19, 756–762. 28199722 10.1093/ntr/ntx046 PMC5896541

[R4] Addicott MA, Sweitzer MM, Froeliger B, Rose JE, McClernon FJ, 2015. Increased functional connectivity in an insula-based network is associated with improved smoking cessation outcomes. Neuropsychopharmacology 40, 2648–2656. 25895453 10.1038/npp.2015.114 PMC4569957

[R5] Amiaz R, Levy D, Vainiger D, Grunhaus L, Zangen A, 2009. Repeated high-frequency transcranial magnetic stimulation over the dorsolateral prefrontal cortex reduces cigarette craving and consumption. Addiction 104, 653–660. 19183128 10.1111/j.1360-0443.2008.02448.x

[R6] Babb S, 2017. Quitting Smoking Among Adults—United States, 2000–2015. MMWR. Morbidity and Mortality Weekly Report 65. 10.15585/mmwr.mm6552a1 28056007

[R7] Barrett LF, Simmons WK, 2015. Interoceptive predictions in the brain. Nature reviews. Neuroscience 16, 419–429. 26016744 10.1038/nrn3950 PMC4731102

[R8] Bi Y, Yuan K, Guan Y, Cheng J, Zhang Y, Li Y, Yu D, Qin W, Tian J, 2017. Altered resting state functional connectivity of anterior insula in young smokers. Brain Imaging Behav. 11, 155–165. 26843002 10.1007/s11682-016-9511-z

[R9] Bickel WK, Odum AL, Madden GJ, 1999. Impulsivity and cigarette smoking: delay discounting in current, never, and ex-smokers. Psychopharmacology 146, 447–454. 10550495 10.1007/pl00005490

[R10] Breese CR, Marks MJ, Logel J, Adams CE, Sullivan B, Collins AC, Leonard S, 1997. Effect of smoking history on [3H]nicotine binding in human postmortem brain. J. Pharmacol. Exp. Ther 282, 7–13. 9223534

[R11] Brunoni AR, Chaimani A, Moffa AH, Razza LB, Gattaz WF, Daskalakis ZJ, Carvalho AF, 2017. Repetitive transcranial magnetic stimulation for the acute treatment of major depressive episodes: a systematic review with network meta-analysis. JAMA Psychiatry 74, 143–152. 28030740 10.1001/jamapsychiatry.2016.3644

[R12] Cauda F, Costa T, Torta DM, Sacco K, D’Agata F, Duca S, Geminiani G, Fox PT, Vercelli A, 2012. Meta-analytic clustering of the insular cortex: characterizing the meta-analytic connectivity of the insula when involved in active tasks. NeuroImage 62, 343–355. 22521480 10.1016/j.neuroimage.2012.04.012 PMC4782788

[R13] Cauda F, D’Agata F, Sacco K, Duca S, Geminiani G, Vercelli A, 2011. Functional connectivity of the insula in the resting brain. NeuroImage 55, 8–23. 21111053 10.1016/j.neuroimage.2010.11.049

[R14] Chanes L, Barrett LF, 2016. Redefining the role of limbic areas in cortical processing. Trends Cogn. Sci 20, 96–106. 26704857 10.1016/j.tics.2015.11.005 PMC4780414

[R15] Chang D, Zhang J, Peng W, Shen Z, Gao X, Du Y, Ge Q, Song D, Shang Y, Wang Z, 2018. Smoking cessation with 20 hz repetitive transcranial magnetic stimulation (rTMS) applied to two brain regions: a pilot study. Front. Hum. Neurosci 12, 344. 30319373 10.3389/fnhum.2018.00344 PMC6166007

[R16] Chang LJ, Yarkoni T, Khaw MW, Sanfey AG, 2013. Decoding the role of the insula in human cognition: functional parcellation and large-scale reverse inference. Cereb. cortex (New York, N.Y.: 1991) 23, 739–749. 10.1093/cercor/bhs065 PMC3563343 22437053

[R17] Claus ED, Blaine SK, Filbey FM, Mayer AR, Hutchison KE, 2013. Association between nicotine dependence severity, BOLD response to smoking cues, and functional connectivity. Neuropsychopharmacology 38, 2363–2372. 23708507 10.1038/npp.2013.134 PMC3799055

[R18] Coll MP, Penton T, Hobson H, 2017. Important methodological issues regarding the use of transcranial magnetic stimulation to investigate interoceptive processing: a Comment on Pollatos et al. (2016). Philos. Trans. R. Soc. Lond. B Biol. Sci 372. 10.1098/rstb.2016.0506 PMC5394648 28396481

[R19] Craig AD, 2002. How do you feel? Interoception: the sense of the physiological condition of the body. Nature reviews. Neuroscience 3, 655–666. 12154366 10.1038/nrn894

[R20] Craig AD, 2009. How do you feel–now? The anterior insula and human awareness. Nature reviews. Neuroscience 10, 59–70. 10.1038/nrn2555 19096369

[R21] Craig AD, 2011. Significance of the insula for the evolution of human awareness of feelings from the body. Ann. N. Y. Acad. Sci 1225, 72–82. 21534994 10.1111/j.1749-6632.2011.05990.x

[R22] Deen B, Pitskel NB, Pelphrey KA, 2011. Three systems of insular functional connectivity identified with cluster analysis. Cereb. cortex (New York, N.Y.: 1991) 21, 1498–1506. 10.1093/cercor/bhq186 PMC3116731 21097516

[R23] Deng ZD, Lisanby SH, Peterchev AV, 2013. Electric field depth-focality tradeoff in transcranial magnetic stimulation: simulation comparison of 50 coil designs. Brain Stimul. 6, 1–13. 22483681 10.1016/j.brs.2012.02.005 PMC3568257

[R24] Dias NR, Peechatka AL, Janes AC, 2016. Insula reactivity to negative stimuli is associated with daily cigarette use: a preliminary investigation using the human connectome database. Drug Alcohol Depend. 159, 277–280. 26748411 10.1016/j.drugalcdep.2015.12.010 PMC4724488

[R25] Dieler AC, Dresler T, Joachim K, Deckert J, Herrmann MJ, Fallgatter AJ, 2014. Can intermittent theta burst stimulation as add-on to psychotherapy improve nicotine abstinence? Results from a pilot study. Eur. Addict. Res 20, 248–253. 24924851 10.1159/000357941

[R26] Ding X, Lee SW, 2013. Changes of functional and effective connectivity in smoking replenishment on deprived heavy smokers: a resting-state FMRI study. PLoS One 8, e59331. 23527165 10.1371/journal.pone.0059331 PMC3602016

[R27] Dinur-Klein L, Dannon P, Hadar A, Rosenberg O, Roth Y, Kotler M, Zangen A, 2014. Smoking cessation induced by deep repetitive transcranial magnetic stimulation of the prefrontal and insular cortices: a prospective, randomized controlled trial. Biol. Psychiatry 76, 742–749. 25038985 10.1016/j.biopsych.2014.05.020

[R28] Dosenbach NU, Fair DA, Miezin FM, Cohen AL, Wenger KK, Dosenbach RA, Fox MD, Snyder AZ, Vincent JL, Raichle ME, Schlaggar BL, Petersen SE, 2007. Distinct brain networks for adaptive and stable task control in humans. Proc. Natl. Acad. Sci. U. S. A 104, 11073–11078. 17576922 10.1073/pnas.0704320104 PMC1904171

[R29] Ferguson SG, Shiffman S, 2009. The relevance and treatment of cue-induced cravings in tobacco dependence. J. Subst. Abuse Treat 36, 235–243. 18715743 10.1016/j.jsat.2008.06.005

[R30] Forget B, Pushparaj A, Le Foll B, 2010. Granular insular cortex inactivation as a novel therapeutic strategy for nicotine addiction. Biol. Psychiatry 68, 265–271. 20299008 10.1016/j.biopsych.2010.01.029

[R31] Gentry CL, Lukas RJ, 2002. Regulation of nicotinic acetylcholine receptor numbers and function by chronic nicotine exposure. Current drug targets. CNS Neurol. Disord 1, 359–385. 10.2174/1568007023339184 12769610

[R32] Gu X, Lohrenz T, Salas R, Baldwin PR, Soltani A, Kirk U, Cinciripini PM, Montague PR, 2016. Belief about nicotine modulates subjective craving and insula activity in deprived smokers. Front. Psychiatry 7, 126. 27468271 10.3389/fpsyt.2016.00126 PMC4942468

[R33] Hahn B, Ross TJ, Yang Y, Kim I, Huestis MA, Stein EA, 2007. Nicotine enhances visuospatial attention by deactivating areas of the resting brain default network. J. Neurosci 27, 3477–3489. 17392464 10.1523/JNEUROSCI.5129-06.2007 PMC2707841

[R34] Hobkirk AL, Nichols TT, Foulds J, Yingst JM, Veldheer S, Hrabovsky S, Richie J, Eissenberg T, Wilson SJ, 2018. Changes in resting state functional brain connectivity and withdrawal symptoms are associated with acute electronic cigarette use. Brain Res. Bull 138, 56–63. 28528203 10.1016/j.brainresbull.2017.05.010 PMC5693791

[R35] Hoogendam JM, Ramakers GM, Di Lazzaro V, 2010. Physiology of repetitive transcranial magnetic stimulation of the human brain. Brain Stimul. 3, 95–118. 20633438 10.1016/j.brs.2009.10.005

[R36] Huang W, King JA, Ursprung WW, Zheng S, Zhang N, Kennedy DN, Ziedonis D, DiFranza JR, 2014. The development and expression of physical nicotine dependence corresponds to structural and functional alterations in the anterior cingulateprecuneus pathway. Brain Behav. 4, 408–417. 24944870 10.1002/brb3.227 PMC4055191

[R37] Jackson KJ, Muldoon PP, De Biasi M, Damaj MI, 2015. New mechanisms and perspectives in nicotine withdrawal. Neuropharmacology 96, 223–234. 25433149 10.1016/j.neuropharm.2014.11.009 PMC4444410

[R38] Janes AC, Farmer S, Peechatka AL, Frederick Bde B, Lukas SE, 2015a. Insuladorsal anterior cingulate cortex coupling is associated with enhanced brain reactivity to smoking cues. Neuropsychopharmacology 40, 1561–1568. 25567427 10.1038/npp.2015.9 PMC4915269

[R39] Janes AC, Gilman JM, Radoman M, Pachas G, Fava M, Evins AE, 2017. Revisiting the role of the insula and smoking cue-reactivity in relapse: a replication and extension of neuroimaging findings. Drug Alcohol Depend. 179, 8–12. 28735078 10.1016/j.drugalcdep.2017.06.012 PMC5599349

[R40] Janes AC, Pizzagalli DA, Richardt S, de BFB, Chuzi S, Pachas G, Culhane MA, Holmes AJ, Fava M, Evins AE, Kaufman MJ, 2010. Brain reactivity to smoking cues prior to smoking cessation predicts ability to maintain tobacco abstinence. Biol. Psychiatry 67, 722–729. 20172508 10.1016/j.biopsych.2009.12.034 PMC2954596

[R41] Janes AC, Ross RS, Farmer S, Frederick BB, Nickerson LD, Lukas SE, Stern CE, 2015b. Memory retrieval of smoking-related images induce greater insula activation as revealed by an fMRI-based delayed matching to sample task. Addict. Biol 20, 349–356. 24261848 10.1111/adb.12112 PMC4031308

[R42] Johann M, Wiegand R, Kharraz A, Bobbe G, Sommer G, Hajak G, Wodarz N, Eichhammer P, 2003. Repetitiv Transcranial Magnetic Stimulation in Nicotine Dependence. Psychiatr. Prax 30, 129–131. 13130356 10.1055/s-2003-39733

[R43] Kelly C, Toro R, Di Martino A, Cox CL, Bellec P, Castellanos FX, Milham MP, 2012. A convergent functional architecture of the insula emerges across imaging modalities. NeuroImage 61, 1129–1142. 22440648 10.1016/j.neuroimage.2012.03.021 PMC3376229

[R44] Kleckner IR, Zhang J, Touroutoglou A, Chanes L, Xia C, Simmons WK, Quigley KS, Dickerson BC, Barrett LF, 2017. Evidence for a large-scale brain system supporting allostasis and interoception in humans. Nat. Hum. Behav 1. 10.1038/s41562-017-0069 PMC5624222 28983518

[R45] Kozak K, Sharif-Razi M, Morozova M, Gaudette EV, Barr MS, Daskalakis ZJ, Blumberger DM, George TP, 2018. Effects of short-term, high-frequency repetitive transcranial magnetic stimulation to bilateral dorsolateral prefrontal cortex on smoking behavior and cognition in patients with schizophrenia and non-psychiatric controls. Schizophr. Res10.1016/j.schres.2018.02.015 PMC6108952 29486960

[R46] Kuhn J, Bauer R, Pohl S, Lenartz D, Huff W, Kim EH, Klosterkoetter J, Sturm V, 2009. Observations on unaided smoking cessation after deep brain stimulation of the nucleus accumbens. Eur. Addict. Res 15, 196–201. 19622886 10.1159/000228930

[R47] Kurth F, Zilles K, Fox PT, Laird AR, Eickhoff SB, 2010. A link between the systems: functional differentiation and integration within the human insula revealed by meta-analysis. Brain Struct. Funct 214, 519–534. 20512376 10.1007/s00429-010-0255-z PMC4801482

[R48] Kutlu MG, Burke D, Slade S, Hall BJ, Rose JE, Levin ED, 2013. Role of insular cortex D(1) and D(2) dopamine receptors in nicotine self-administration in rats. Behav. Brain Res 256, 273–278. 23948214 10.1016/j.bbr.2013.08.005 PMC3874126

[R49] Lerman C, Gu H, Loughead J, Ruparel K, Yang Y, Stein EA, 2014. Large-scale brain network coupling predicts acute nicotine abstinence effects on craving and cognitive function. JAMA Psychiatry 71, 523–530. 24622915 10.1001/jamapsychiatry.2013.4091 PMC4097018

[R50] Li X, Du L, Sahlem GL, Badran BW, Henderson S, George MS, 2017a. Repetitive transcranial magnetic stimulation (rTMS) of the dorsolateral prefrontal cortex reduces resting-state insula activity and modulates functional connectivity of the orbitofrontal cortex in cigarette smokers. Drug Alcohol Depend. 174, 98–105. 28319755 10.1016/j.drugalcdep.2017.02.002 PMC5400684

[R51] Li X, Hartwell KJ, Owens M, Lematty T, Borckardt JJ, Hanlon CA, Brady KT, George MS, 2013. Repetitive transcranial magnetic stimulation of the dorsolateral prefrontal cortex reduces nicotine cue craving. Biol. Psychiatry 73, 714–720. 23485014 10.1016/j.biopsych.2013.01.003 PMC3615051

[R52] Li X, Sahlem GL, Badran BW, McTeague LM, Hanlon CA, Hartwell KJ, Henderson S, George MS, 2017b. Transcranial magnetic stimulation of the dorsal lateral prefrontal cortex inhibits medial orbitofrontal activity in smokers. Am. J. Addict 26, 788–794. 28898485 10.1111/ajad.12621 PMC5699931

[R53] Li Y, Yuan K, Guan Y, Cheng J, Bi Y, Shi S, Xue T, Lu X, Qin W, Yu D, Tian J, 2017c. The implication of salience network abnormalities in young male adult smokers. Brain Imaging Behav. 11, 943–953. 27437925 10.1007/s11682-016-9568-8

[R54] Malik S, Jacobs M, Cho SS, Boileau I, Blumberger D, Heilig M, Wilson A, Daskalakis ZJ, Strafella AP, Zangen A, Le Foll B, 2017. Deep TMS of the insula using the H-coil modulates dopamine release: a crossover [(11)C] PHNO-PET pilot trial in healthy humans. Brain Imaging Behav. 10.1007/s11682-017-9800-1 29170944

[R55] Menon V, 2011. Large-scale brain networks and psychopathology: a unifying triple network model. Trends Cogn. Sci 15, 483–506. 21908230 10.1016/j.tics.2011.08.003

[R56] Milev RV, Giacobbe P, Kennedy SH, Blumberger DM, Daskalakis ZJ, Downar J, Modirrousta M, Patry S, Vila-Rodriguez F, Lam RW, MacQueen GM, Parikh SV, Ravindran AV, 2016. Canadian network for mood and anxiety treatments (CANMAT) 2016 clinical guidelines for the management of adults with major depressive disorder: section 4. Neurostimulation treatments. Can. J. Psychiatry 61, 561–575. 27486154 10.1177/0706743716660033 PMC4994792

[R57] Moran-Santa Maria MM, Hartwell KJ, Hanlon CA, Canterberry M, Lematty T, Owens M, Brady KT, George MS, 2015. Right anterior insula connectivity is important for cue-induced craving in nicotine-dependent smokers. Addict. Biol 20, 407–414. 24529072 10.1111/adb.12124 PMC4133311

[R58] Moran LV, Sampath H, Stein EA, Hong LE, 2012. Insular and anterior cingulate circuits in smokers with schizophrenia. Schizophr. Res 142, 223–229. 23021898 10.1016/j.schres.2012.08.033 PMC3502674

[R59] Mutschler I, Wieckhorst B, Kowalevski S, Derix J, Wentlandt J, Schulze-Bonhage A, Ball T, 2009. Functional organization of the human anterior insular cortex. Neurosci. Lett 457, 66–70. 19429164 10.1016/j.neulet.2009.03.101

[R60] Naqvi NH, Rudrauf D, Damasio H, Bechara A, 2007. Damage to the insula disrupts addiction to cigarette smoking. Science 315, 531–534. 17255515 10.1126/science.1135926 PMC3698854

[R61] Nestler E, Hyman S, Holtzman D, Malenka R, 2015. Molecular Neuropharmacology: A Foundation for Clinical Neuroscience. McGraw-Hill.

[R62] Nestor L, McCabe E, Jones J, Clancy L, Garavan H, 2011. Differences in “bottom-up” and “top-down” neural activity in current and former cigarette smokers: evidence for neural substrates which may promote nicotine abstinence through increased cognitive control. NeuroImage 56, 2258–2275. 21440645 10.1016/j.neuroimage.2011.03.054

[R63] Nieuwenhuys R, 2012. The insular cortex: a review. Prog. Brain Res 195, 123–163. 22230626 10.1016/B978-0-444-53860-4.00007-6

[R64] Pollatos O, Herbert BM, Mai S, Kammer T, 2016. Changes in interoceptive processes following brain stimulation. Philos. Trans. R. Soc. Lond. B Biol. Sci 371. 10.1098/rstb.2016.0016 PMC5062104 28080973

[R65] Pollatos O, Kammer T, 2017. Reply to Coll et al.’ Important methodological issues regarding the use of transcranial magnetic stimulation to investigate interoceptive processing’ (2017). Philos. Trans. R. Soc. Lond. B Biol. Sci 372. 10.1098/rstb.2017.0046 PMC5394650 28396483

[R66] Pripfl J, Tomova L, Riecansky I, Lamm C, 2014. Transcranial magnetic stimulation of the left dorsolateral prefrontal cortex decreases cue-induced nicotine craving and EEG delta power. Brain Stimul. 7, 226–233. 24468092 10.1016/j.brs.2013.11.003

[R67] Pushparaj A, Hamani C, Yu W, Shin DS, Kang B, Nobrega JN, Le Foll B, 2013. Electrical stimulation of the insular region attenuates nicotine-taking and nicotine-seeking behaviors. Neuropsychopharmacology 38, 690–698. 23249816 10.1038/npp.2012.235 PMC3572467

[R68] Salling MC, Martinez D, 2016. Brain stimulation in addiction. Neuropsychopharmacology 41, 2798–2809. 27240657 10.1038/npp.2016.80 PMC5061891

[R69] Seeley WW, Menon V, Schatzberg AF, Keller J, Glover GH, Kenna H, Reiss AL, Greicius MD, 2007. Dissociable intrinsic connectivity networks for salience processing and executive control. J. Neurosci 27, 2349–2356. 17329432 10.1523/JNEUROSCI.5587-06.2007 PMC2680293

[R70] Shannon CE, 1948. A mathematical theory of communication. Bell Syst. Tech. J 27, 379–423.

[R71] Sheffer CE, Bickel WK, Brandon TH, Franck CT, Deen D, Panissidi L, Abdali SA, Pittman JC, Lunden SE, Prashad N, Malhotra R, Mantovani A, 2018. Preventing relapse to smoking with transcranial magnetic stimulation: feasibility and potential efficacy. Drug Alcohol Depend. 182, 8–18. 29120861 10.1016/j.drugalcdep.2017.09.037 PMC5836507

[R72] Stead LF, Lancaster T, 2012. Combined pharmacotherapy and behavioural interventions for smoking cessation. Cochrane Database Syst. Rev 10, Cd008286. 23076944 10.1002/14651858.CD008286.pub2

[R73] Suner-Soler R, Grau A, Gras ME, Font-Mayolas S, Silva Y, Davalos A, Cruz V, Rodrigo J, Serena J, 2012. Smoking cessation 1 year poststroke and damage to the insular cortex. Stroke; J. Cereb. Circ 43, 131–136. 10.1161/STROKEAHA.111.630004 22052507

[R74] Sutherland MT, McHugh MJ, Pariyadath V, Stein EA, 2012. Resting state functional connectivity in addiction: lessons learned and a road ahead. NeuroImage 62, 2281–2295. 22326834 10.1016/j.neuroimage.2012.01.117 PMC3401637

[R75] Sutherland MT, Ray KL, Riedel MC, Yanes JA, Stein EA, Laird AR, 2015. Neurobiological impact of nicotinic acetylcholine receptor agonists: an activation likelihood estimation meta-analysis of pharmacologic neuroimaging studies. Biol. Psychiatry 78, 711–720. 25662104 10.1016/j.biopsych.2014.12.021 PMC4494985

[R76] Tanabe J, Nyberg E, Martin LF, Martin J, Cordes D, Kronberg E, Tregellas JR, 2011. Nicotine effects on default mode network during resting state. Psychopharmacology 216, 287–295. 21331518 10.1007/s00213-011-2221-8 PMC3486925

[R77] Thielscher A, Antunes A, Saturnino GB, 2015. Field modeling for transcranial magnetic stimulation: a useful tool to understand the physiological effects of TMS? Conference proceedings : … annual International Conference of the IEEE engineering in medicine and biology society. IEEE Engineering in Medicine and Biology Society. Annual Conference 2015. pp. 222–225. 10.1109/EMBC.2015.7318340 26736240

[R78] Tobacco TCPGT, 2008. A clinical practice guideline for treating tobacco use and dependence: 2008 update: a US public health service report. Am. J. Prev. Med 35, 158. 18617085 10.1016/j.amepre.2008.04.009 PMC4465757

[R79] Tombu MN, Asplund CL, Dux PE, Godwin D, Martin JW, Marois R, 2011. A Unified attentional bottleneck in the human brain. Proc. Natl. Acad. Sci. U. S. A 108, 13426–13431. 21825137 10.1073/pnas.1103583108 PMC3158154

[R80] Trautwein FM, Singer T, Kanske P, 2016. Stimulus-driven reorienting impairs executive control of attention: evidence for a common bottleneck in anterior insula. Cereb. Cortex (New York, N.Y. : 1991). 10.1093/cercor/bhw225 PMC5066828 27550866

[R81] Trojak B, Meille V, Achab S, Lalanne L, Poquet H, Ponavoy E, Blaise E, Bonin B, Chauvet-Gelinier JC, 2015. Transcranial magnetic stimulation combined with nicotine replacement therapy for smoking cessation: a randomized controlled trial. Brain Stimul. 8, 1168–1174. 26590478 10.1016/j.brs.2015.06.004

[R82] Uddin LQ, 2015. Salience processing and insular cortical function and dysfunction. Nature reviews. Neuroscience 16, 55–61. 10.1038/nrn3857 25406711

[R83] Uddin LQ, Kinnison J, Pessoa L, Anderson ML, 2014. Beyond the tripartite cognition-emotion-interoception model of the human insular cortex. J. Cogn. Neurosci 26, 16–27. 23937691 10.1162/jocn_a_00462 PMC4074004

[R84] Ussher M, Beard E, Abikoye G, Hajek P, West R, 2013. Urge to smoke over 52 weeks of abstinence. Psychopharmacology 226, 83–89. 23052572 10.1007/s00213-012-2886-7

[R85] Volkow ND, Wang GJ, Fowler JS, Tomasi D, 2012. Addiction circuitry in the human brain. Annu. Rev. Pharmacol. Toxicol 52, 321–336. 21961707 10.1146/annurev-pharmtox-010611-134625 PMC3477468

[R86] Wang Y, Zhu L, Zou Q, Cui Q, Liao W, Duan X, Biswal B, Chen H, 2018. Frequency dependent hub role of the dorsal and ventral right anterior insula. NeuroImage 165, 112–117. 28986206 10.1016/j.neuroimage.2017.10.004

[R87] Wilcox CE, Calhoun VD, Rachakonda S, Claus ED, Littlewood RA, Mickey J, Arenella PB, Hutchison KE, 2017. Functional network connectivity predicts treatment outcome during treatment of nicotine use disorder. Psychiatry Res. 265, 45–53. 10.1016/j.pscychresns.2017.04.011 PMC5522183 28525877

[R88] Wing VC, Bacher I, Wu BS, Daskalakis ZJ, George TP, 2012. High frequency repetitive transcranial magnetic stimulation reduces tobacco craving in schizophrenia. Schizophr. Res 139, 264–266. 22464727 10.1016/j.schres.2012.03.006

[R89] Wu T, Wang X, Wu Q, Spagna A, Yang J, Yuan C, Wu Y, Gao Z, Hof PR, Fan J, 2019. Anterior insular cortex is a bottleneck of cognitive control. NeuroImage. 10.1016/j.neuroimage.2019.02.042 PMC6550348 30798012

[R90] Wylie KP, Rojas DC, Tanabe J, Martin LF, Tregellas JR, 2012. Nicotine increases brain functional network efficiency. NeuroImage 63, 73–80. 22796985 10.1016/j.neuroimage.2012.06.079 PMC3429645

[R91] Zanchi D, Brody AL, Montandon ML, Kopel R, Emmert K, Preti MG, Van De Ville D, Haller S, 2015. Cigarette smoking leads to persistent and dose-dependent alterations of brain activity and connectivity in anterior insula and anterior cingulate. Addict. Biol 20, 1033–1041. 26303184 10.1111/adb.12292

[R92] Zhou S, Xiao D, Peng P, Wang SK, Liu Z, Qin HY, Li SS, Wang C, 2017. Effect of smoking on resting-state functional connectivity in smokers: an fMRI study. Respirology (Carlton, Vic.) 22, 1118–1124. 28374936 10.1111/resp.13048

